# The impact of residents' knowledge and perception of marine sports tourism destinations on post-COVID-19 tourism attitudes: the mediating effect of tourism acceptance and moderating effect of place identity

**DOI:** 10.3389/fspor.2025.1616394

**Published:** 2025-11-14

**Authors:** Jeongmyeong Song, Kwon-Hyuk Jeong

**Affiliations:** 1 Department of Sport Welfare, Kangnam University, Yongin-si, Gyeonggi-do, Republic of Korea; 2 College of Physical Education, Kyunghee University, Yongin-si, Gyeonggi-do, Republic of Korea

**Keywords:** marine sports tourism, tourism attitudes, place identity, tourism acceptance, COVID-19, residents' perception

## Abstract

This study examines residents' attitudes toward marine sports tourism in the post-COVID-19 era, focusing on the mediating effect of tourism acceptance and the moderating effect of place identity. Data were collected from 231 residents who had lived in marine sports tourism destinations in South Korea for more than 5 years. The results revealed that residents' tourism knowledge and perceptions positively influenced their tourism attitudes. Tourism acceptance demonstrated a significant mediating effect between residents' tourism knowledge, destination perception, and tourism attitudes. Furthermore, place identity exhibited a positive moderating effect on these relationships. The findings suggest that for sustainable development of marine sports tourism destinations, it is essential to establish customized tourism development policies that consider residents' levels of tourism knowledge while emphasizing positive impacts and minimizing negative impacts. Additionally, measures to enhance residents' tourism acceptance and implement policies that consider place identity during the tourism development process are necessary. This study provides valuable insights for marine sports tourism destination managers in promoting sustainable tourism through effective communication with local residents. Future research recommendations include conducting qualitative interviews with residents, implementing pilot studies, and regularly evaluating residents' attitudes as the COVID-19 situation evolves.

## 1 Introduction

The COVID-19 pandemic significantly impacted global tourism, with international tourism expected to decrease by approximately 80% in 2020 (1, 2). Marine sports tourism destinations experienced unique challenges and opportunities for recovery during this unprecedented crisis (3). This study examines how residents' knowledge and perceptions of tourism influence their attitudes toward tourism development in Korean marine sports tourism destinations during the post-pandemic period through the lens of social exchange theory (SET).

This research adopts SET as its core theoretical framework, recognizing that residents' attitudes toward tourism fundamentally stem from their evaluation of exchanges with the tourism industry (4). SET posits that individuals engage in exchanges when perceived benefits exceed costs, and this principle has been widely applied to explain resident–tourist relationships (5). However, the pandemic context and marine sports tourism's unique characteristics necessitate an extended SET framework that incorporates new exchange dimensions and boundary conditions.

For conceptual clarity, this study distinguishes between related but distinct concepts. Marine tourism encompasses all recreational activities in coastal and marine environments, including beach relaxation, coastal sightseeing, and cruise tourism (6, 7). Marine sports tourism, a subset of marine tourism, specifically involves “travel away from one’s primary residence for participating in or view[ing] marine sport activities” (8). This includes active participation in water-based sports such as surfing, sailing, scuba diving, windsurfing, kayaking, and sport fishing, as well as passive consumption through spectating at marine sport events (9). The “sport” aspect distinguishes these activities by their physical skill requirements, competitive elements, and specialized equipment needs (10, 11).

Sport tourism research has established robust theoretical frameworks for understanding the intersection of sport and tourism (12, 13). Gibson (10) identified three primary domains of sport tourism: active sport tourism (traveling to participate), event sport tourism (traveling to watch), and nostalgia sport tourism (visiting sport-related attractions). For marine sports tourism, the active participation domain predominates, characterized by tourists seeking physical engagement with marine environments through structured sporting activities (8). The unique characteristics of marine sports tourism include dependency on natural resources, seasonal variations, specialized skill requirements, and higher risk perceptions compared with land-based sport tourism (14).

Marine environments offer distinct benefits for human well-being. Evidence suggests a positive relationship between blue spaces—areas with substantial inland and coastal waters—and mental health (15, 16). In the context of marine sports tourism, these psychological benefits are enhanced through active physical engagement, combining the therapeutic effects of blue spaces with the well-documented health benefits of sport participation (17). While extensive research has examined terrestrial green spaces such as urban parks and forests (18, 19), studies on blue spaces remain comparatively limited (20, 21). This gap extends to marine sports tourism research, where the intersection of sport, tourism, and marine environments remains underexplored (14). These marine environments have become particularly relevant during the pandemic recovery phase (22), as destinations have sought to leverage their natural assets for sustainable tourism development (23).

In response to the pandemic's impact, destinations have been implementing diverse recovery strategies. According to the Organization for Economic Co-operation and Development (OECD) (1), these efforts include promoting domestic tourism through extended holiday weekends (as in Costa Rica), introducing long-term stay visas for remote workers (Barbados' “Welcome Stamp”), and developing niche markets such as ecotourism and wellness tourism (Thailand's strategy). For marine sports tourism destinations specifically, recovery initiatives have focused on leveraging blue space benefits for mental health promotion (24), implementing strict safety protocols for water-based activities (2), and developing contactless service technologies (25).

The pandemic's impact on tourism has been severe. Following initial declines due to health fears and lockdown measures (26), the emergence of “revenge tourism” marked a shift in travel patterns (27). However, this recovery presented a dilemma for residents of tourism destinations. While recognizing tourism's economic importance, they harbored concerns about health risks and quality of life impacts (3). This tension underscores the importance of understanding residents' attitudes for sustainable tourism development (28–30).

This study also introduces precise conceptual distinctions for key constructs. Tourism acceptance, distinct from general tourism attitudes or support, refers to “residents' willingness to receive and interact with tourists in their community, reflecting both behavioral intentions and emotional readiness” [adapted from (31)]. While tourism attitudes encompass overall evaluative judgments about tourism impacts (positive or negative), and tourism support indicates political or economic backing for development (32), tourism acceptance specifically captures the interpersonal dimension of resident–tourist relationships. This distinction is crucial in the post-pandemic context, where health concerns may create dissonance between recognizing tourism's economic benefits (positive attitudes) and willingness to welcome tourists (acceptance).

Residents' attitudes toward tourism significantly influence destination success, as positive resident–tourist interactions enhance visitor satisfaction and destination reputation (33, 34). Getz (35) established that the social impacts of tourism fundamentally shape resident–tourist relationships, identifying key factors including cultural commodification, demonstration effects, and community cohesion. Building on this foundation, subsequent research has demonstrated that residents' perceptions of these social impacts directly influence their support for tourism development (36, 37).

Social exchange theory (SET) has traditionally explained these attitudes, suggesting residents support tourism when perceived benefits exceed costs (4, 5). The theory posits that residents evaluate tourism based on economic benefits such as job creation and increased income, as well as costs including crowding, inflation, and cultural disruption (38–40). However, SET's assumption of voluntary participation has limitations, as many resident–tourist interactions occur involuntarily (37, 41).

Place identity theory provides an alternative framework, proposing that residents' self-concept related to their location shapes tourism attitudes (42). Research demonstrates that place identity elements—distinctiveness, continuity, self-esteem, and self-efficacy—significantly influence residents' responses to tourism development (43–45). As Getz (46) noted, cultural items such as festivals, music, dance, and rituals are frequently subjected to commodification, with rewards becoming “monetary and divorced from their cultural meaning,” which can affect residents' place-based identity. In post-disaster contexts, place identity positively affects perceived community resilience (47, 48), suggesting its relevance for understanding post-pandemic tourism attitudes.

Despite extensive research on residents' tourism attitudes (49–51), limited attention has focused on Korean marine sports tourism destinations in the post-COVID-19 context. Korea's marine sports tourism sector encompasses diverse activities, including sailing, windsurfing, jet skiing, and sea kayaking, concentrated in coastal regions such as Gangwon Province, which hosts international surfing competitions and marine sports festivals (52). This gap is particularly significant given Korea's substantial marine tourism sector, which attracted over 100 million visitors annually before the pandemic (53). Understanding the complex interplay between residents' tourism knowledge, perceptions, and the social impacts of tourism development is essential for creating sustainable recovery strategies (54).

Tourism acceptance measurement in this study operationalizes the concept through three dimensions: (1) willingness to welcome tourists to the community, (2) readiness to interact with tourists in daily life, and (3) acceptance of tourism-related changes in the community (55). This multidimensional approach distinguishes acceptance from unidimensional constructs such as support, providing a more nuanced understanding of residents' behavioral intentions toward tourist presence.

Therefore, this study aims to (1) examine the impact of residents' tourism knowledge and perceptions on their tourism attitudes in Korean marine sports tourism destinations, (2) investigate the mediating effect of tourism acceptance on these relationships, and (3) analyze the moderating role of place identity. The findings will provide theoretical insights into post-pandemic tourism dynamics and practical guidance for destination managers seeking to foster sustainable tourism development through enhanced community engagement (2, 56).

## 2 Literature review

### 2.1 Social exchange theory in tourism contexts

Social exchange theory provides the foundational framework for understanding resident–tourist relationships. According to SET, social behavior results from exchange processes where individuals seek to maximize benefits and minimize costs (57, 58). In tourism contexts, residents evaluate their support for tourism based on perceived exchanges of resources, where benefits might include economic gains, cultural enrichment, and infrastructure improvements, while costs encompass crowding, environmental degradation, and cultural disruption (4).

The application of SET to tourism has evolved from simple cost–benefit analyses to more sophisticated frameworks incorporating multiple exchange dimensions. Perdue et al. (59) demonstrated that residents' perceptions of tourism impacts directly relate to their willingness to engage in exchanges with the tourism industry. Subsequent research has identified various resources exchanged in tourism contexts, including economic capital, social capital, cultural resources, and environmental quality (60).

### 2.2 Extended SET for post-pandemic marine sports tourism

The pandemic and marine sports tourism contexts necessitate extending traditional SET frameworks through three key dimensions. The COVID-19 pandemic introduced health risks as a critical cost in tourism exchanges. Unlike traditional costs that are primarily economic or sociocultural, health risks represent existential threats that fundamentally alter exchange calculations. Residents must now evaluate whether economic benefits justify potential exposure to health hazards, creating a new dimension in the exchange equation (28). This health risk dimension represents a paradigm shift in how residents calculate exchange outcomes, as existential threats cannot be easily compensated through economic gains alone.

Furthermore, we conceptualize tourism acceptance as residents' behavioral intention to engage in exchanges with tourists, distinct from general attitudes or political support. Within SET, acceptance represents the behavioral manifestation of positive exchange evaluations—residents who perceive favorable exchange outcomes demonstrate greater willingness to welcome tourists, interact in daily life, and accommodate tourism-related changes [adapted from (31)]. This behavioral dimension addresses a gap in traditional SET applications, which have focused predominantly on cognitive evaluations while underexploring how these evaluations translate into actual exchange behaviors. The distinction is crucial because residents may cognitively recognize tourism's benefits while remaining behaviorally reluctant to engage with tourists, particularly in post-pandemic contexts where health concerns create cognitive–behavioral dissonance.

Additionally, place identity functions as a boundary condition within the exchange framework. Residents with strong place identity evaluate tourism exchanges through the lens of community preservation, intensifying their assessment of how tourism affects their place-based self-concept (42). This represents a sociopsychological dimension of exchange evaluation where identity preservation becomes a valued resource in the exchange equation, suggesting that not all exchanges are evaluated through purely rational cost–benefit calculations (43). Place identity introduces non-economic values into the exchange process, creating a filter through which all tourism impacts are evaluated relative to their effects on community character and residents' sense of self.

### 2.3 Tourism acceptance concept

Our extended SET model proposes that residents' tourism attitudes result from complex exchange evaluations that integrate these new dimensions. Knowledge serves as an exchange resource that enables accurate assessment of outcomes, allowing residents to make informed decisions about the true costs and benefits of tourism (61). When residents possess greater knowledge about tourism operations, impacts, and dynamics, they can more precisely calculate exchange outcomes, reducing uncertainty and enabling more confident exchange decisions (62). Perception represents the subjective interpretation of these costs and benefits, filtered through individual and community experiences. These perceptions are not merely cognitive assessments but emotionally laden evaluations shaped by personal experiences, community narratives, and cultural values (63).

Acceptance manifests as the behavioral willingness to engage in tourist exchanges, bridging cognitive evaluations and actual behaviors. This behavioral component is critical because it represents the translation of abstract exchange calculations into concrete actions—welcoming tourists, providing assistance, sharing local knowledge, and tolerating tourism-related inconveniences (64). Place identity operates as a moderating lens through which all exchanges are evaluated, potentially strengthening or weakening the influence of rational assessments based on identity-protection motivations (65). Residents with strong place identity may reject economically beneficial exchanges if they threaten community character or embrace economically marginal exchanges if they reinforce place-based values (51).

This integrated framework explains all hypothesized relationships through exchange mechanisms while accounting for the unique characteristics of post-pandemic marine sports tourism contexts. The framework recognizes that marine sports tourism creates specific exchange dynamics—residents must share coastal resources, tolerate noise from water activities, and accept safety risks from amateur participants (14). The pandemic overlay adds health considerations to these calculations (66), while place identity influences how residents weigh preservation of their maritime heritage against tourism development opportunities (44). By integrating these elements within an extended SET framework, we provide a comprehensive theoretical foundation for understanding how residents form attitudes toward marine sports tourism in the contemporary context.

### 2.4 Residents' place identity in sport tourism destinations

The theoretical foundations of place identity draw from environmental psychology and human geography. Proshansky (67) initially conceptualized place identity as a sub-structure of self-identity, comparable to social identity but focused on the relationship between self and physical settings. This framework has evolved to recognize place identity as comprising four key components: distinctiveness (what makes a place unique), continuity (connection between past and present), self-esteem (pride derived from place association), and self-efficacy (belief in one's ability to fulfill goals within that place) (42, 68).

In tourism contexts, place identity has emerged as a critical factor influencing residents' responses to tourism development. Gu and Ryan (43) demonstrated that residents with strong place identity evaluate tourism through the lens of heritage preservation and community character maintenance. Their seminal study in Beijing's hutongs revealed that place identity elements—particularly distinctiveness and continuity—significantly shaped residents' tourism attitudes beyond economic considerations. This finding challenges purely economic models of resident attitudes, suggesting that identity-based evaluations operate through different cognitive pathways.

Recent empirical studies have expanded the understanding of place identity's role in tourism contexts. Strzelecka et al. (65) demonstrated that place identity affects residents' empowerment perceptions, with strongly attached residents more likely to engage in tourism planning processes. Their research revealed that place identity operates as both a motivational force and an evaluative framework, influencing not only attitudes but also behavioral intentions toward tourism participation. Similarly, Eusébio et al. (44) found that place identity in island destinations created unique dynamics where residents' maritime heritage connections influenced their tolerance for marine tourism activities.

In summary, place identity represents a multifaceted construct that profoundly influences how residents perceive, evaluate, and respond to tourism development. Its role extends beyond simple attachment to encompass complex identity processes that filter tourism impacts through the lens of self-concept and community meanings. Understanding place identity's influence on tourism attitudes requires recognizing its dynamic, culturally specific, and multidimensional nature, particularly in specialized contexts such as marine sports tourism destinations.

### 2.5 Recent social exchange theory studies

Social exchange theory applications have become more sophisticated, moving beyond simple cost–benefit analysis to incorporate emotional and cultural factors. Recent research demonstrates the need for multi-theoretical approaches and cultural sensitivity. Gaonkar and Sukthankar (69) proposed a revised SET framework, expanding beyond traditional cost–benefit analysis to include community attachment, involvement, perceived cultural impact, and attitudes toward cultural tourism/tourists. Their empirical findings show that community attachment and involvement significantly influence support through perceived cultural impacts. Ward and Berno (70) integrated SET with the contact hypothesis and integrated threat theory, showing that SET alone is insufficient to explain resident attitudes toward tourists. Their research reveals intercultural contact frequency, perceived threat levels, and stereotypes significantly predict attitudes beyond economic considerations.

### 2.6 Sport tourism residents' attitudes: theoretical framework

Social exchange theory remains the dominant framework, but with growing recognition of the need for integrated approaches. Recent research has developed comprehensive frameworks combining multiple theoretical perspectives. Kim et al. (71) developed a six-factor model of perceived social impacts with validated dimensions: economic benefits, community pride, community development, economic costs, traffic problems, and security risks. The theoretical contribution provides a validated multidimensional scale for sport tourism event impacts.

González-García et al. (72) created a multidimensional scale measuring economic, social, cultural, environmental, and political–administrative impacts. Their research shows residents' perceptions vary across impact dimensions, with support depending on perceived benefits vs. costs. For marine sports tourism specifically, Gon et al. (73) applied social representation theory to nautical tourism, finding residents cluster into supporters (51%), cautious (29%), and skeptics (20%), with a long tradition influencing positive attitudes. Recent 2023 research applies the destination social responsibility (DSR) model to marine sports tourism, showing DSR positively affects destination identification and environmentally responsible behavior (74).

## 3 Research hypotheses

### 3.1 The relationship between tourism knowledge and tourism attitudes

Tourism knowledge represents residents' factual understanding of tourism's mechanisms, impacts, and operations within their community. This cognitive resource serves as a foundation for attitude formation by enabling residents to make informed evaluations of tourism development (75). The knowledge–attitude relationship has received substantial empirical support across diverse tourism contexts. Tosun et al. (76) conducted a rigorous structural equation modeling (SEM) analysis with 484 residents in Seville, Spain, demonstrating that tourism knowledge significantly affects residents' perceptions of economic impacts. Their findings reveal that knowledge operates through domain-specific pathways, particularly influencing economic impact perceptions. This suggests that residents with greater tourism knowledge develop a more sophisticated understanding of tourism's economic mechanisms.

However, the relationship demonstrates complexity beyond simple positive associations. Vidal Rua (77) found that tourism knowledge relates positively to both perceived positive and negative impacts in Girona, Spain, using SEM with strong reliability measures. The study revealed that “less knowledgeable residents are aware about the benefits of tourism but not very informed about its negative impacts,” aligning with critical citizen theory (78).

In marine tourism contexts, Masud et al. (79) examined 310 residents in Malaysian marine protected areas, confirming significant knowledge–attitude relationships specific to coastal environments. The unique characteristics of marine sports tourism—including specialized equipment requirements, weather dependency, and distinct risk profiles—may intensify the role of knowledge in attitude formation. Therefore, the following hypothesis was established.

**Hypothesis 1:** Residents' knowledge of tourism positively influences their tourism attitudes in marine sports tourism destinations.

### 3.2 The relationship between tourism perception and tourism attitudes

Tourism perception encompasses residents' subjective interpretation of tourism's multidimensional impacts on their community, including economic, sociocultural, and environmental dimensions. According to social exchange theory, residents evaluate tourism based on perceived benefits vs. costs, with this evaluative process fundamentally shaping their attitudes (4). The COVID-19 pandemic context has added new dimensions to perception–attitude relationships. Hao et al. (22) showed that risk perception negatively affects tourism attitudes, while crisis communication effectiveness maintains positive relationships. These findings suggest perception–attitude links remain stable but incorporate new evaluative criteria during crisis periods. In marine sports tourism destinations, perception takes on unique characteristics. Residents must evaluate marine-specific factors such as beach congestion, water quality changes, noise from motorized water sports, and safety concerns related to amateur participants. These specialized perceptions likely create stronger attitude formation processes. Therefore, the following hypothesis was established.

**Hypothesis 2:** Residents' perception of tourism positively influences their tourism attitudes in marine sports tourism destinations.

### 3.3 The relationship between tourism knowledge and tourism acceptance

Tourism acceptance, conceptualized as residents' behavioral willingness to receive and interact with tourists, represents a distinct construct from general attitudes or political support (31). The relationship between knowledge and acceptance operates through uncertainty reduction mechanisms, where increased understanding diminishes anxiety about tourist presence.

Shen et al. (80) examined 370 residents in Huangshan, finding that place image (a knowledge-related construct) affects attitudes, which then predict pro-tourism behavioral intentions. Mediation analysis revealed that place image indirectly affects behavioral intention through attitudes.

Disaster tourism research provides additional insights. Hao et al. (81) demonstrated that disaster knowledge directly predicts behavioral intentions, indicating that factual understanding reduces uncertainty and increases willingness to engage with tourism. In marine sports contexts, technical knowledge about safety procedures, environmental protocols, and activity requirements likely reduces residents' anxiety about tourist presence. Therefore, the following hypothesis was established.

**Hypothesis 3:** Residents' knowledge of tourism positively influences their tourism acceptance in marine sports tourism destinations.

### 3.4 The relationship between tourism perception and tourism acceptance

The relationship between perception and acceptance operates through evaluative consistency mechanisms. When residents perceive tourism positively, they become more willing to engage in welcoming behaviors, creating alignment between cognitive evaluations and behavioral intentions. Li et al. (82) provided clear evidence that residents' participation (a form of acceptance) partially mediates the relationship between perceived benefits and support for tourism in Guilin. This suggests that positive perceptions facilitate acceptance behaviors through motivational pathways. The Botswana COVID-19 study (83) found that 67.5% of respondents indicated willingness to accept inconvenience for tourism benefits, demonstrating how positive economic perceptions translate into acceptance behaviors even during crisis periods. In marine sports tourism contexts, perceptions of environmental benefits or economic opportunities likely influence residents' willingness to share coastal resources. Therefore, the following hypothesis was established.

**Hypothesis 4:** Residents' perception of tourism positively influences their tourism acceptance in marine sports tourism destinations.

### 3.5 The relationship between tourism acceptance and tourism attitudes

While traditional models position attitudes as antecedents to behavior, emerging evidence suggests that behavioral experiences can reshape attitudinal positions through experiential learning processes. Tourism acceptance behaviors may influence subsequent attitude formation through direct interaction experiences. Woosnam and Lee (84) demonstrated that emotional solidarity with tourists significantly predicts tourism support, with 48% of residents' attitudes toward tourism development predicted by emotional solidarity factors. The behavioral components of solidarity—such as welcoming interactions and sympathetic understanding—suggest that acceptance behaviors influence attitude formation through emotional pathways. The Botswana study (83) found that 84.4% of respondents were willing to welcome tourists due to economic contributions, with this behavioral willingness preceding positive attitude reinforcement. In marine sports tourism contexts, acceptance behaviors involve specific interactions such as sharing beach access and providing local knowledge, which likely create cognitive dissonance reduction processes. Therefore, the following hypothesis was established.

**Hypothesis 5:** Tourism acceptance positively influences tourism attitudes in marine sports tourism destinations.

### 3.6 The mediating effect of tourism acceptance: the relationship between tourism knowledge and tourism attitudes

The theoretical framework positions tourism acceptance as a critical mediating mechanism linking cognitive evaluations to attitude formation. This mediation represents a behavioral pathway where knowledge first influences willingness to interact with tourists, which subsequently shapes overall tourism attitudes. Shen et al. (80) support this mediation, showing indirect effects of cognitive factors on behavioral intentions through attitudinal pathways. Residents with greater tourism knowledge experience reduced uncertainty, increasing their willingness to engage in accepting behaviors. These positive interaction experiences then reinforce favorable attitudes through behavioral confirmation processes.

In marine sports tourism destinations, this mediation may be particularly pronounced due to the technical nature of activities requiring local knowledge exchange. Therefore, the following hypothesis was established.

**Hypothesis 6:** Tourism acceptance mediates the relationship between residents' knowledge of tourism and their tourism attitudes in marine sports tourism destinations.

### 3.7 The mediating effect of tourism acceptance: the relationship between tourism perception and tourism attitudes

The perception–acceptance–attitude mediation operates through evaluative consistency mechanisms. When residents perceive tourism's benefits as outweighing costs, they become more willing to engage in welcoming behaviors, which then strengthen tourism attitudes through positive feedback loops. Li et al. (82) empirically demonstrated this mediation, with participation mediating perception-support relationships. The study confirmed that positive perceptions facilitate acceptance behaviors, which subsequently enhance tourism support through experiential reinforcement. In marine sports tourism contexts, residents who perceive positive impacts become more willing to share resources and interact with tourists, creating positive experiences that validate initial perceptions. Therefore, the following hypothesis was established.

**Hypothesis 7:** Tourism acceptance mediates the relationship between residents' perception of tourism and their tourism attitudes in marine sports tourism destinations.

### 3.8 The moderating effect of place identity: the relationship between residents' knowledge of tourism and residents' attitudes toward tourism

Place identity represents residents' self-concept derived from their physical environment and emotional connections to place-based meanings (85). This psychological construct serves as an interpretive lens that may strengthen or weaken the knowledge–attitude relationship. Wang and Chen (42) demonstrated that place-based self-esteem and self-efficacy affect how residents process tourism impacts, with length of residence moderating these relationships. Residents with stronger place identity show more protective responses to tourism development, evaluating impacts through the lens of community preservation. In marine sports tourism destinations, residents with strong coastal identity may process tourism knowledge differently, viewing it either as validation of their maritime heritage or as threat-related information. This identity-based processing likely amplifies knowledge effects when tourism aligns with place meanings. Therefore, the following hypothesis was established.

**Hypothesis 8:** Place identity moderates the relationship between residents' knowledge of tourism and their tourism attitudes, such that the positive relationship is stronger when place identity is high.

### 3.9 The moderating effect of place identity: the relationship between residents' perception of tourism and residents' attitudes toward tourism

Place identity also moderates how perceptions translate into attitudes by influencing the weight residents assign to different impact dimensions. Residents with strong place attachment may prioritize community preservation over economic benefits in their evaluative processes.

Li et al. (86) found that place attachment negatively moderates relationships between residents' perceptions and tourism attitudes, suggesting that strongly attached residents maintain consistent attitudes regardless of changing perceptions. This protective mechanism may reflect identity-based resistance to external influences.

However, in marine sports tourism contexts where activities align with coastal lifestyle values, place identity may amplify positive perception–attitude relationships. Residents who identify strongly with marine environments may view compatible tourism as reinforcing their place-based identity. Therefore, the following hypothesis was established.

**Hypothesis 9:** Place identity moderates the relationship between residents' perception of tourism and their tourism attitudes, such that the positive relationship is stronger when place identity is high.

### 3.10 Research model

**Figure 1:**
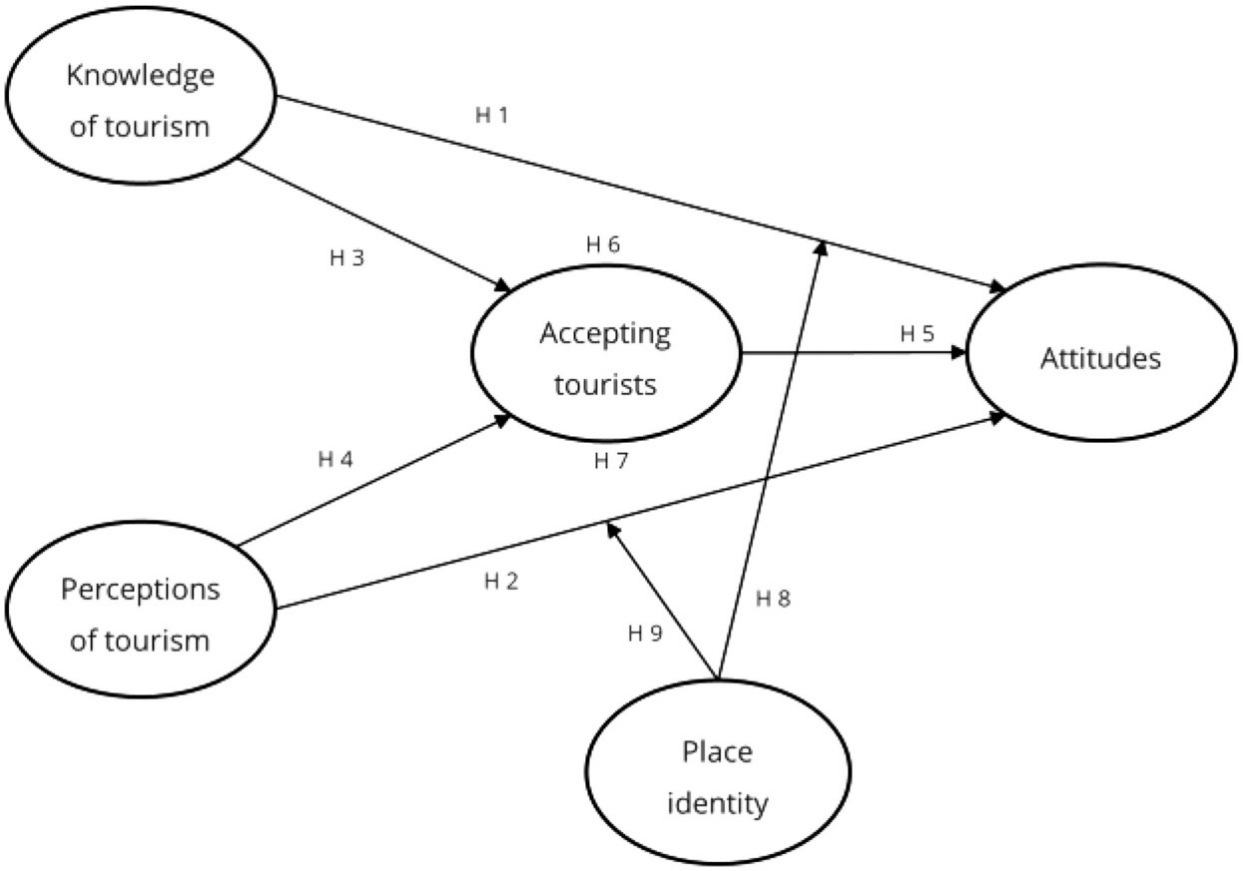
The conceptual model.

**Figure 2:**
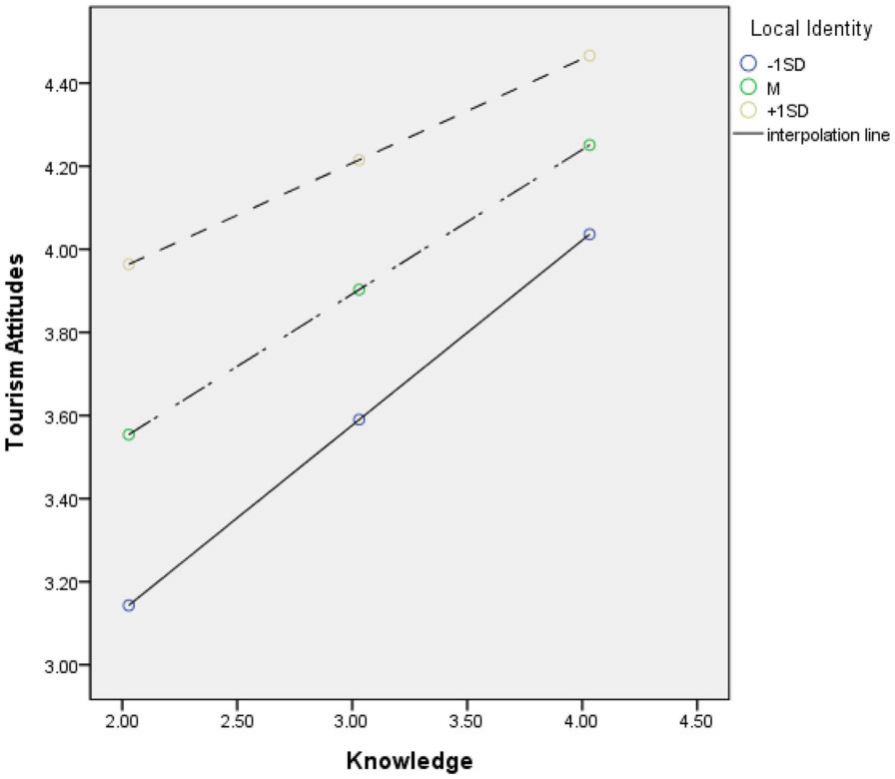
Interaction plot of knowledge of tourism at different levels of place attachment.

Two conceptual models were developed based on previous studies, as illustrated in Figures 1 and 2. Model 1 examines the mediating effect of tourist receptivity on the relationship between the independent and dependent variables after COVID-19, and Model 2 describes the moderating effect of regional identity on the relationship between the independent and dependent variables after COVID-19. The independent variables are “knowledge of tourism” and “perception of tourism” of residents of the tourist area, and the dependent variable is “attitudes of residents of the tourist area.”

## 4 Methodology

### 4.1 Research design

This study employed a cross-sectional survey design to examine the relationships between residents' tourism knowledge, perceptions, acceptance, and attitudes in marine sports tourism destinations. A quantitative approach was adopted using structural equation modeling (SEM) to test the proposed hypotheses, including mediating effects of tourism acceptance and moderating effects of place identity. This research design was selected to capture the complex interrelationships among multiple variables simultaneously and to provide generalizable findings across the marine sports tourism context in Gangwon Province, South Korea.

### 4.2 Study setting and population

The study was conducted in three major marine sports tourism destinations in Gangwon Province: Yangyang (surfing), Gangneung (sailing), and Sokcho (diving). These locations were strategically selected to represent diverse marine sports activities and different scales of tourism development. The target population comprised permanent residents aged 18 years or older who had lived in these communities for a minimum of 5 years. The 5-year residence requirement ensured participants had sufficient experience with both pre-pandemic and pandemic-era tourism impacts, enabling informed responses about tourism dynamics in their communities.

### 4.3 Sampling techniques

A multistage sampling approach was employed to enhance representativeness while acknowledging practical constraints. First, purposive sampling was used to select the three study locations based on their prominence in different marine sports activities. Second, within each location, convenience sampling with strategic recruitment measures was implemented to maximize demographic diversity.

Sample size was determined using G*Power 3.1 software, indicating a minimum of 200 participants for SEM analysis with anticipated effect sizes (*f*^2^ = 0.15), statistical power of 0.80, and significance level of 0.05. To account for potential incomplete responses and ensure adequate representation across the three locations, a target sample of 350 participants was established.

### 4.4 Data collection methods

Data collection was conducted over a 2-month period from 30 October 2024 to 30 December 2024, utilizing an online self-administered questionnaire distributed through Naver Forms, a widely used survey platform in Korea. The selection of this platform was deliberate, as its familiarity among Korean respondents reduced potential technical barriers to participation and its mobile-friendly interface accommodated the increasing prevalence of mobile internet usage in Korea.

A comprehensive multichannel recruitment strategy was implemented to maximize reach and demographic diversity. Online recruitment utilized several digital platforms, including local community Facebook groups with combined memberships exceeding 12,000 residents, KakaoTalk open chat rooms dedicated to local residents, and Naver Café platforms for each destination area. These online channels were complemented by physical distribution methods, with QR code posters strategically placed in high-traffic community locations, including community centers, local government offices, and other public spaces frequented by residents. To ensure representation across different demographic groups, data collection was deliberately scheduled at various times of day—morning, afternoon, and evening—and across different days of the week, capturing residents with diverse work schedules and lifestyles.

The questionnaire design prioritized both data quality and participant experience. It began with carefully constructed screening questions to verify participant eligibility, including confirmation of at least 5 years of residence, primary residence status in the study area, and age verification of 18 years or older. The average completion time ranged from 15 to 20 min, a duration that balanced comprehensive data collection needs with respect for participants' time. To maintain response authenticity and minimize participation solely for rewards, no direct monetary incentives were provided. Instead, participants were offered non-monetary benefits including access to a summary report of research findings upon study completion and entry into a raffle for local marine sports activity vouchers. Ten vouchers worth 50,000 KRW each were offered as raffle prizes, with this incentive disclosed only at the survey's conclusion to prevent reward-motivated participation that might compromise response quality. Table 1 presents the detailed demographic characteristics of the study participants, providing a comprehensive overview of the sample composition.

**Table 1:** Demographic characteristics of the study participants.

Variable	Category	*N*	%
Gender	Male	176	59.7
	Female	119	40.3
Age	20s	33	11.2
	30s	74	25.1
	40s	109	36.9
	50s	40	13.6
	60s and above	39	13.2
Education Level	Less than middle school	14	4.7
	High school graduate	28	9.5
	University graduate	222	75.3
	Master's degree or above	23	7.8
	Doctorate	8	2.7
Monthly Income	<990,000 KRW	40	13.6
	1,000,000 KRW	33	11.2
	2,000,000 KRW	73	24.7
	3,000,000 KRW	62	21.0
	4,000,000 KRW	38	12.9
	5,000,000 KRW or more	49	16.6
Total			295

### 4.5 Research instruments

The research instrument consisted of a structured questionnaire incorporating validated scales carefully adapted from established tourism research. Each scale was selected based on its theoretical relevance, previous validation in similar contexts, and appropriateness for the marine sports tourism setting.

Tourism knowledge was measured using three items adapted from Zhu and Deng (87), designed to capture residents' factual understanding of tourism's mechanisms and impacts in their community. These items assessed awareness of tourism situations, understanding of tourism risk causes, and knowledge about tourism-related information. Tourism perception was similarly measured with three items from the same source, focusing on residents' subjective evaluations of how COVID-19 had affected their community through tourism channels, including economic stagnation, unemployment rates, and diminished tourism development.

Tourism attitudes were assessed using three items adapted from Peters et al. (88), measuring residents' overall evaluative judgments toward tourism development. These items captured general openness to further tourism development, support for new forms of post-COVID tourism, and willingness to support tourism development despite pandemic challenges. Tourism acceptance, conceptualized as behavioral willingness rather than mere attitudinal support, was measured through three items adapted from Ashraf et al. (55). These items specifically addressed willingness to accept tourists after COVID-19, acceptance of domestic tourists, and conditional acceptance based on spatial separation between tourist and residential areas.

Place identity, the most complex construct in the study, was measured using six items adapted from Wang and Xu (51). This scale captured multiple dimensions of residents' emotional connections to their marine sports destination, including memory evocation, environmental reminiscence, pride in residence, personal identification with place praise, meaningfulness of place, and emotional responses to media criticism of their community.

All items employed 5-point Likert scales ranging from strongly disagree (1) to strongly agree (5), a format familiar to Korean respondents and appropriate for capturing gradations in attitudes and perceptions. Prior to the main data collection, the instrument underwent rigorous pre-testing with 30 residents from the target communities. This pretest phase revealed the need for minor wording adjustments to enhance clarity and cultural appropriateness, particularly in translating concepts related to place identity and tourism acceptance into locally meaningful terms.

### 4.6 Validity and reliability

To verify the validity of each variable, we conducted an exploratory factor analysis was conducted. The exploratory factor analysis of the constructs—knowledge of tourism, effect on the region, attitude toward tourism, accepting tourists, and place attachment—resulted in the extraction of five distinct concepts. The Kaiser–Meyer–Olkin (KMO) measure of sampling adequacy value was 0.861, meeting the recommended threshold. The cumulative variance explained by the five factors was 76.530% (Table 2).

**Table 2:** Exploratory factor analysis results of online information characteristics.

Item	Place attachment	Knowledge of tourism	effect on the region	Attitude toward tourism	Accepting tourists
This marine sports region always evokes strong memories for me	0.855	0.095	0.116	0.217	0.073
The environment of this marine sports region always reminds me of my past	0.832	−0.042	0.101	0.163	0.081
Living in this marine sports region makes me feel very proud	0.806	0.084	0.223	0.163	0.191
When someone praises this marine sports region, it feels like a personal compliment to me	0.775	0.010	0.331	0.119	0.058
This marine sports region is very meaningful to me	0.729	0.025	0.095	0.050	0.305
If a story in the media criticizes this marine sports region, I feel embarrassed	0.684	0.318	0.346	−0.053	−0.071
I am concerned about travel information (i.e., the travel situation)	0.036	0.853	0.121	0.168	0.253
I know about the causes of tourism risks	0.131	0.828	0.080	0.198	0.176
I know about the situations of tourism risks	0.000	0.826	−0.112	0.155	0.227
I think the economic stagnation arising in this marine sports region has been caused by the effects of COVID-19	0.251	0.167	0.825	0.218	0.069
I think the rate of unemployment in this marine sports region is increasing because of the effects of COVID-19	0.287	−0.063	0.796	0.268	0.161
I think tourism development in this marine sports region is diminishing due to the effects of COVID-19	0.342	−0.009	0.743	0.276	0.230
Generally, I am open to further tourism development	0.088	0.153	0.227	0.799	0.177
I support a new form of tourism after the spread of COVID-19	0.249	0.292	0.232	0.782	0.062
I support tourism development even with COVID-19	0.215	0.206	0.248	0.759	0.282
Accepting tourists after COVID-19	0.147	0.238	0.154	0.124	0.818
I will accept tourists from around Korea	0.122	0.227	0.085	0.208	0.813
I accept tourists if the tourist areas are separated from residential areas, including shops for local people	0.215	0.405	0.164	0.150	0.612
Eigenvalue	4.154	2.671	2.438	2.313	2.199
% of variance	23.079	14.841	13.543	12.850	12.217
Cumulative %	23.079	37.920	51.463	64.313	76.530
* Cronbach’s α*	*0.* *904*	0.873	0.880	0.862	0.815

KMO = 0.861, Bartlett's *χ*^2^ = 79.833 (*p* < 0.001), df = 153.

Note: Values in italics represent the results of exploratory factor analysis, including eigenvalues, percentage of variance explained by each factor, cumulative percentage of variance, and Cronbach's alpha reliability coefficients.

### 4.7 Data analysis strategies

The data analysis strategy was designed as a systematic, multistage process utilizing IBM SPSS Statistics 28 for preliminary analyses and AMOS 26 for structural equation modeling. This comprehensive approach ensured thorough examination of data quality, measurement properties, and hypothesis testing.

The initial phase focused on data screening and preparation. Missing data patterns were examined to determine whether data were missing completely at random, missing at random, or missing not at random, with appropriate treatment strategies applied accordingly. Outlier detection employed Mahalanobis distance calculations to identify multivariate outliers that might unduly influence results. Normality assessment examined skewness and kurtosis values for all variables, with the criteria of skewness within ±2 and kurtosis within ±4 indicating acceptable univariate normality for subsequent analyses.

Descriptive and preliminary analyses provided a foundational understanding of the data. Demographic characteristics were analyzed using frequency distributions to profile the sample composition. Descriptive statistics including means, standard deviations, and ranges were calculated for all study variables to understand their central tendencies and variability. Correlation analysis examined bivariate relationships among variables, providing initial insights into hypothesized relationships and checking for potential multicollinearity issues.

Measurement model validation proceeded through both exploratory and confirmatory phases. Exploratory factor analysis using principal component analysis with varimax rotation assessed the dimensionality of constructs and identified any cross-loading items. This was followed by confirmatory factor analysis to validate the measurement model's structure, with careful attention to modification indices and theoretical justification for any model refinements. Reliability testing ensured all constructs exceeded the minimum Cronbach's alpha threshold of 0.7, while validity assessment examined both convergent validity through average variance extracted and discriminant validity through comparison of squared correlations with average variance extracted values.

Structural model testing employed sophisticated analytical techniques appropriate for the complexity of the hypothesized relationships. Direct relationships specified in Hypotheses 1 through 5 were tested using structural equation modeling, which allowed simultaneous examination of multiple relationships while accounting for measurement error. Mediation analyses for Hypotheses 6 and 7 utilized Hayes' PROCESS macro Model 4 with 5,000 bootstrap samples to generate bias-corrected confidence intervals for indirect effects. Moderation analyses for Hypotheses 8 and 9 employed PROCESS macro Model 1, with particular attention to plotting interaction effects for interpretation. Throughout structural model testing, multiple fit indices were examined including chi-square to degrees of freedom ratio (acceptable if <3), comparative fit index (acceptable if >0.90), and root mean square error of approximation (acceptable if <0.08).

Post-hoc analyses extended beyond hypothesis testing to provide additional insights. Multigroup analysis examined whether structural relationships differed across the three study locations, potentially revealing location-specific dynamics in marine sports tourism contexts. Common method bias, a potential concern in single-source survey data, was assessed using Harman's single-factor test to ensure that variance in the data was not primarily attributable to the measurement method rather than the constructs of interest.

### 4.8 Ethical considerations

Ethical considerations were paramount throughout the research process, beginning with formal approval from the Institutional Review Board of Kangnam University (Protocol number: KNU-HR2409002). This approval process ensured that the study design, recruitment procedures, and data handling protocols met rigorous ethical standards for research involving human participants.

Informed consent procedures were carefully implemented to ensure voluntary participation and full understanding of the research purpose and procedures. Before accessing the survey questions, all participants were presented with detailed information about the study's objectives, the voluntary nature of participation, data confidentiality measures, and their rights as research participants. Participants actively indicated their consent by proceeding with the survey after reading this information. The consent process emphasized that participants could withdraw from the study at any time without penalty and that incomplete surveys would be deleted from the dataset.

Data confidentiality measures were comprehensive and multilayered. The online survey platform was configured to collect responses anonymously, with no personally identifiable information recorded beyond basic demographic categories. IP addresses were used only for duplicate prevention and were not stored with survey responses. All data were transferred to secure, password-protected storage immediately upon collection, with access limited to the research team. The presentation of results in aggregate form further ensured that no individual participant could be identified from published findings.

### 4.9 Limitations and quality control

Quality control measures were implemented throughout the data collection process to ensure the integrity and reliability of the dataset. The online survey platform's capabilities were leveraged to prevent duplicate responses through IP address monitoring, although this was balanced with allowing multiple household members to participate using different devices. Response time monitoring identified surveys completed in <5 min, which were flagged for additional scrutiny as this duration was insufficient for thoughtful response to all items. Attention check questions were strategically embedded within the questionnaire to identify participants who were not reading questions carefully. Pattern response detection algorithms identified cases of straight-lining, where participants selected the same response option across multiple consecutive items regardless of item content.

The data cleaning process was systematic and transparent. From the initial 350 responses collected, 32 were excluded for failing to meet the 5-year residence criterion despite passing initial screening, suggesting some misunderstanding or misrepresentation during the eligibility check. An additional 19 responses were removed due to substantial missing data that exceeded acceptable thresholds for imputation. Four responses showed clear evidence of pattern responding, with identical responses across conceptually opposite items, indicating a lack of engagement with survey content. This rigorous cleaning process yielded 295 valid responses, representing an 84.3% retention rate that exceeds typical expectations for online survey research.

The data cleaning process was systematic and transparent. From the initial 350 responses collected, 32 were excluded for failing to meet the 5-year residence criterion despite passing initial screening, suggesting some misunderstanding or misrepresentation during the eligibility check. An additional 19 responses were removed due to substantial missing data that exceeded acceptable thresholds for imputation. Four responses showed clear evidence of pattern responding, with identical responses across conceptually opposite items, indicating a lack of engagement with survey content. This rigorous cleaning process yielded 295 valid responses, representing an 84.3% retention rate that exceeds typical expectations for online survey research. The final sample demonstrated reasonable demographic diversity that aligned with known population characteristics of the study areas. The gender distribution showed 59.7% male participants, consistent with the demographic structure of Korean coastal communities, where marine-related industries traditionally employ more males. Age distribution achieved representation across all adult life stages, from young adults in their 20s (11.2%) through seniors in their 60s and above (13.2%), with the largest group being those in their 40s (36.9%), reflecting the working-age population concentration in these communities. Educational attainment in the sample ranged from less than middle school completion (4.7%) through doctoral degrees (2.7%), with the majority holding university degrees (75.3%). This distribution corresponds with South Korea's high educational attainment rates while still capturing educational diversity. Monthly income levels showed substantial variation, from <990,000 KRW (13.6%) to over 5,000,000 KRW (16.6%), representing the full economic spectrum of residents in these communities. The sample composition regarding tourism business interests proved particularly relevant for this study. Approximately half of the participants reported having tourism-related business interests, while the other half had no direct economic stake in tourism. This balanced distribution enables examination of attitudes across different economic relationships with tourism, avoiding potential bias toward those with direct financial interests in the industry. The diversity across all demographic dimensions suggests that the multichannel recruitment strategy successfully reached various resident groups within the marine sports tourism destinations.

## 5 Results

### 5.1 Descriptive analysis

This study estimated the measurement and structural models using the maximum likelihood method. To verify the univariate normality, the skewness and kurtosis of the variables were analyzed.

As shown in Table 3, the skewness values ranged from −1.548 to 0.133, and the kurtosis values ranged from −0.507 to 3.472. These results meet the criteria of skewness within ±2 and kurtosis within ±4, as recommended by West et al. (89). Therefore, the normality of the data was established.

**Table 3:** Descriptive statistics analysis by factor.

Variable	*N*	M	Sd	Skewness		Kurtosis	
				M	SD	M	SD
Knowledge of tourism	231	3.030	1.002	0.133	0.160	−0.507	0.319
Effect on the region	231	4.345	0.722	−1.548	0.160	3.472	0.319
Attitude toward tourism	231	3.882	0.926	−0.948	0.160	0.942	0.319
Accepting tourists	231	3.566	0.801	−0.296	0.160	0.454	0.319
Place attachment	231	4.161	0.662	−0.602	0.160	0.200	0.319

### 5.2 Results of correlation analysis

To examine the relationships among the variables established in the research, a Pearson product–moment correlation analysis was conducted. The results of the correlation analysis are presented in Table 4. The analysis revealed statistically significant correlations among the variables at the *p* < 0.01 level. This indicates that the study constructs were significantly associated with one another. Furthermore, an examination of the inter-factor correlations showed that the relationships among the lower-order factors were also statistically significant. However, the magnitudes of the correlations were all below the multicollinearity threshold of 0.8, suggesting that multicollinearity was not a concern in the dataset.

**Table 4:** Results of inter-factor correlation analysis.

	Knowledge of tourism	Effect on the region	Attitude toward tourism	Accepting tourists	Place attachment
Knowledge of tourism	1				
Effect on the region	0.194**	1			
Attitude toward tourism	0.457**	0.581**	1		
Accepting tourists	0.567**	0.401**	0.507**	1	
Place attachment	0.215**	0.584**	0.431**	0.384**	1

** *p* < 0.01.

### 5.3 Causal relationships between variables

Table 5 presents the results of the multiple regression analysis conducted to examine the influence of knowledge of tourism and effect on the region on attitude toward tourism.

**Table 5:** Results of multiple regression analysis on the influence of knowledge of tourism and effect on the region on attitude toward tourism.

Dependent variable	Independent variables	*β*	SE	*β*	*t*	VIF
Attitude toward tourism	(Constant)	0.031	0.288		0.106	
	Knowledge of tourism	0.331	0.046	0.358	7.226***	1.039
	Effect on the region	0.656	0.064	0.511	10.313***	1.039
*R* = 0.679, *R*^2^ = 0.461, adjusted *R*^2^ = 0.456, *F* = 97.384***						

***p < 0.001.

The analysis revealed that the regression model was statistically significant at the 0.001 level (*F* = 97.384), indicating that knowledge of tourism and effect on the region had a significant influence on attitude toward tourism. The model accounted for 45.6% of the variance in the dependent variable (adjusted *R*^2^ = 0.4,564).

Furthermore, the variance inflation factor (VIF) values were all below 10, suggesting that multicollinearity was not a concern in the regression model.

Examining the specific beta coefficients, the analysis showed that effect on the region (*β* = 0.246) and knowledge of tourism (*β* = 0.225) were both significant predictors of attitude toward tourism at the 0.05 level. This implies that as perceptions of effect on the region and knowledge of tourism increased, attitude toward tourism also tended to increase.

### 5.4 Mediating effect of tourism destination personality

To investigate the mediating effect of accepting tourists on the relationships between knowledge of tourism, effect on the region, and attitude toward tourism, a PROCESS macro (model 4) was utilized. The bootstrap sample size was set to 5,000, and the confidence interval was 95%. The analysis results are presented in Table 6.

**Table 6:** Direct and indirect effects analysis: tourism knowledge and regional effects on tourism attitudes.

Dependent variable	Independent variables		Coefficients	SE	*t*	*p*	LLCI	ULCI
Attitude toward tourism	Knowledge of tourism	Mediating variable model (dependent variable: accepting tourists)						
		Constant	2.192	0.139	15.786	0.000	1.919	2.466
		Knowledge of tourism	0.453	0.044	10.415	0.000	0.368	0.539
		Dependent variable model (dependent variable: attitude toward tourism)						
		Constant	1.675	0.236	7.094	0.000	1.210	2.140
		Knowledge of tourism	0.231	0.062	3.716	0.000	0.109	0.353
		Accepting tourists	0.423	0.078	5.435	0.000	0.269	0.576
	Effect on the region	Mediating variable model (dependent variable: accepting tourists)						
		constant	1.632	0.296	5.519	0.000	1.050	2.215
		Effect on the region	0.445	0.067	6.626	0.000	0.313	0.577
		Dependent variable model (dependent variable: attitude toward tourism)						
		Constant	0.030	0.301	0.099	0.921	−0.564	0.624
		Effect on the region	0.576	0.070	8.213	0.000	0.438	0.715
		Accepting tourists	0.378	0.063	5.976	0.000	0.253	0.503

As shown in Table 7, the results showed that knowledge of tourism had a significant positive effect on attitude toward tourism (*β* = 0.231, *p* < 0.000), and Accepting Tourists had a significant positive effect on attitude toward tourism (*β* = 0.423, *p* < 0.000). This indicates that accepting tourists mediated the relationship between knowledge of tourism and attitude toward tourism. The total effect of knowledge of tourism on attitude toward tourism was *β* = 0.423 (*p* < 0.000), but when the mediator accepting tourists was introduced, the direct effect of knowledge of tourism on attitude toward tourism decreased to *β* = 0.231 (*p* < 0.000), suggesting a partial mediation effect.

**Table 7:** Mediation analysis: tourist acceptance as a mediator between tourism knowledge, regional effects, and tourism attitudes.

Dependent variable	Independent variable	Effect	Effect	Boot SE	Boot LLCI	Boot ULCI
Attitude toward tourism	Knowledge of tourism	Total effect	0.423	0.054	0.316	0.530
		Direct effect	0.231	0.062	0.109	0.353
		Indirect	0.192	0.052	0.098	0.302
	Effect on the region	Total effect	0.744	0.069	0.609	0.880
		Direct effect	0.576	0.070	0.438	0.715
		Indirect	0.168	0.043	0.090	0.259

Secondly, the analysis revealed that effect on the region had a significant positive effect on attitude toward tourism (*β* = 0.576, *p* < 0.000), and accepting tourists had a significant positive effect on attitude toward tourism (*β* = 0.378, *p* < 0.000). This indicates that accepting tourists mediated the relationship between effect on the region and attitude toward Tourism. The total effect of effect on the region on attitude toward tourism was *β* = 0.744 (*p* < 0.000), but when the mediator accepting tourists was introduced, the direct effect of effect on the region on attitude toward tourism decreased to *β* = 0.576 (*p* < 0.000), again suggesting a partial mediation effect.

Furthermore, the bootstrapping results for the indirect effects showed that the 95% confidence intervals did not contain zero for all the examined relationships, confirming the statistical significance of the mediation effects.

### 5.5 Analysis of place attachment's moderating effect on tourism knowledge, regional effects, and tourism attitudes

The study aimed to verify the moderating effect of place attachment on the relationship between knowledge of tourism and attitude toward tourism using the PROCESS macro's Model 1. The analysis results are presented in Tables 8, 9.

**Table 8:** Interaction effect analysis: tourism knowledge and place attachment.

Category	*β*	SE	*t*	*p*	LLCI	ULCI
Constant	−0.975	0.882	−1.105	0.270	−2.714	0.764
Knowledge of tourism	0.961	0.288	3.337	0.001	0.393	1.528
Place attachment	0.919	0.215	4.269	0.000	0.495	1.343
Knowledge of tourism × place attachment	−0.147	0.069	−2.144	0.033	−0.283	−0.012
*R*^2^ = 339, *F* = 0.38.724***						
−1SD	0.445	0.067	6.668	0.000	0.314	0.577
M	0.348	0.051	6.808	0.000	0.247	0.449
+1SD	0.251	0.070	3.584	0.000	0.113	0.389

***p < 0.001.

**Table 9:** Interaction effect analysis: regional effects and place attachment.

Category	*β*	SE	*t*	*p*	LLCI	ULCI
Constant	−2.241	0.914	−2.452	0.015	−4.043	−0.440
Effect on the region	1.132	0.215	5.268	0.000	0.709	1.555
Place attachment	1.123	0.281	3.994	0.000	0.569	1.678
Effect on the region × place attachment	−0.183	0.063	−2.913	0.004	−0.307	−0.059
*R*^2^ = 0.439, *F* = 59.175***						
−1SD	0.598	0.075	7.947	0.000	0.450	0.746
M	0.426	0.083	5.123	0.000	0.262	0.590
+1SD	0.254	0.123	2.069	0.040	0.012	0.497
Bootstrapping (5,000 resamples)						

***p < 0.001.

First, the analysis of the moderating effect of place attachment on the relationship between knowledge of tourism and attitude toward tourism revealed a statistically significant interaction between knowledge of tourism and place attachment (*β* = −0.147, *t* = −2.144, *p* < 0.05). Following the recommendation of Aiken and West (90), we examined the conditional effect through bootstrapping to explore the interaction further. The results showed that the lower and upper limits of the 95% confidence interval for the conditional effect of knowledge of tourism on attitude toward tourism did not include zero at −1SD (*β* = 0.445, *t* = 6.668, *p* < 0.001), M (*β* = 0.348, *t* = 6.808, *p* < 0.001), and +1SD (*β* = 0.251, *t* = 3.584, *p* > 0.001) of place attachment (91). This indicates that the moderating effect of place attachment is statistically significant.

Specifically, when place attachment is at −1SD, a one-unit increase in knowledge of tourism leads to a 0.577 increase in attitude toward tourism. When place attachment is at the mean, a one-unit increase in knowledge of tourism leads to a 0.449 increase in attitude toward tourism. When place attachment is at +1SD, a one-unit increase in knowledge of tourism leads to a 0.389 increase in attitude toward tourism. The graphical representation of the moderating effect is presented in Figure 2 using the pick-a-point approach.

**Figure 3:**
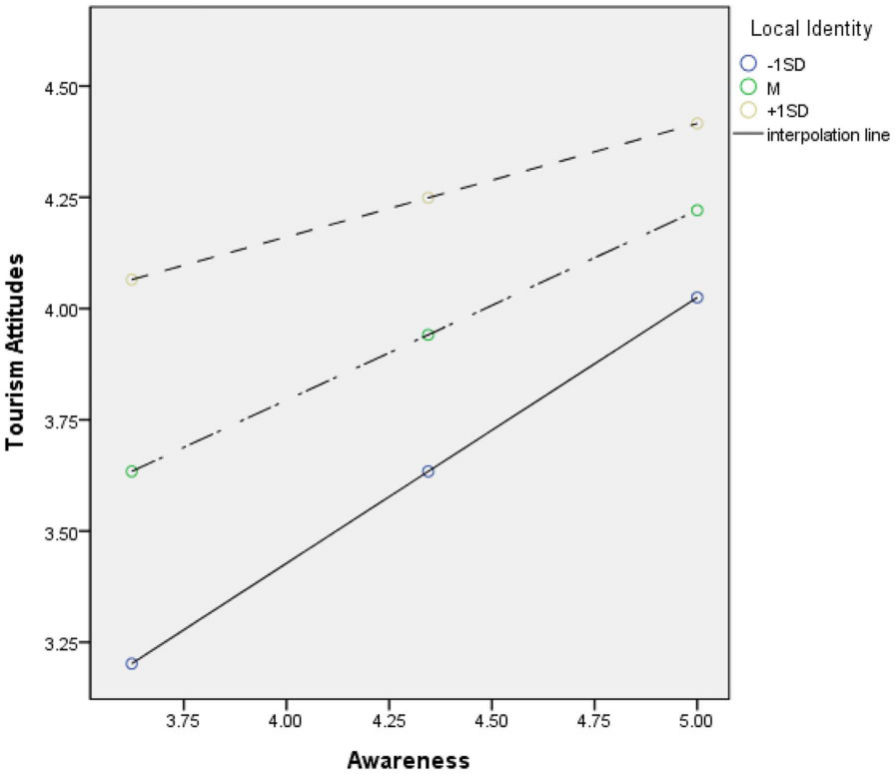
Interaction plot of effect on the region at different levels of place attachment.

Second, analysis of the moderating effect of place attachment on the relationship between effect on the region and attitude toward tourism revealed that the interaction term between effect on the region and place attachment was statistically significant (*β* = −0.183, *t* = −2.913, *p* < 0.01). Following Aiken and West's (90) recommendation, conditional effects were examined through bootstrapping to explore the interaction in detail. The results indicated that the moderating effect of place attachment on the relationship between effect on the region and attitude toward tourism was statistically significant at −1SD (*β* = 0.598, *t* = 7.947, *p* < 0.001), M (*β* = 0.426, *t* = 5.123, *p* < 0.01), and +1SD (*β* = 0.254, *t* = 2.069, *p* < 0.05), as the lower limit confidence interval (LLCI) and upper limit confidence interval (ULCI) did not contain zero (91). The pick-a-point approach was employed to illustrate the moderating effect, as shown in Figure 3.

## 6 Discussion

This study aims to examine the mediating effect of tourism acceptance and the moderating effect of place identity in the relationship between marine sports tourism destination knowledge and perception on tourism attitudes among local residents. Based on the analysis results, the following discussion is presented.

First, the tourism knowledge of residents in marine sports tourism destinations was found to have a positive impact on their tourism attitudes. In this regard, knowledge has been established as a crucial resource for local residents within the social exchange theory (SET) framework, serving as a determinant of their position within social exchange networks (61). Nunkoo (92), Nunkoo and So (93), and Vidal Rua (77) reported that while tourism knowledge does not explain perceptions of positive impacts, it is associated with perceptions of negative impacts. These researchers provided a compelling explanation that knowledge reinforces the notion of “critical citizens” who maintain a more critical stance toward additional tourism development (78).

According to Vidal Rua (77), residents with greater knowledge can serve as gatekeepers for sustainable tourism development in their region, and this knowledge enhances their concept of empowerment (94). For instance, Joo et al. (94) reported that the more knowledge local residents possess about tourism, the greater psychological, social, and political empowerment they feel. Conversely, lack of knowledge has been identified as a significant barrier to residents' participation in tourism-related decision-making processes (95). These findings confirm that residents' tourism knowledge is a crucial antecedent in forming tourism attitudes in marine sports tourism destinations. From the perspective of social exchange theory, this empirically demonstrates that higher levels of knowledge enable better understanding and evaluation of tourism-related benefits and costs. Furthermore, considering the distinctive characteristics of marine sports tourism, specialized tourism knowledge plays an even more significant role in fostering positive tourism attitudes.

Second, residents' perceptions of tourism in marine sports tourism destinations were found to have a positive impact on their tourism attitudes. Previous studies have indicated that residents in tourism destinations are aware that tourism increases living costs (62, 96–98), leading to price increases in goods and services sold in tourist areas (99, 102). Generally, while the influx of tourists improves residents' living standards, it simultaneously leads to inflation (103), resulting in increased real estate values and housing prices (59, 99, 102, 104, 105), as well as land values (98, 99, 102). The overall assessment of these impacts tends to be positive, as residents acknowledge that the tourism industry enriches the community's structure (60). Moreover, numerous studies have indicated that economic benefits are the most highly valued and sought-after element by local residents (103, 106, 107).

Economic benefits have been shown to significantly influence residents' attitudes toward tourism (100, 108, 109), as many residents perceive tourism as improving, benefiting, and growing the local economy (59, 110). Consequently, almost all studies examining the relationship between economic benefits and attitudes toward tourism have reported positive correlations (40, 59, 111–114). As an exception, Johnson et al. (101) found that residents perceived the tourism industry as one that provides low-wage and low-quality employment opportunities.

These findings confirm that residents' tourism perceptions are a key determinant in forming tourism attitudes in marine sports tourism destinations. This supports the existing social exchange theory, which posits that positive perceptions of tourism impacts lead to favorable tourism attitudes. Furthermore, this study empirically demonstrates that in the context of marine sports tourism, balanced perceptions of economic, sociocultural, and environmental impacts are crucial.

Third, tourism acceptance was found to have a positive mediating effect on the relationship between residents' tourism knowledge, destination perception, and tourism attitudes in marine sports tourism destinations. In this regard, Woosnam (31) empirically verified that emotional solidarity is a key factor in explaining residents' attitudes toward tourism and tourism development. Particularly, higher levels of emotional solidarity were found to increase support for tourism development. McGehee and Andereck (62) demonstrated that tourism knowledge functions as a major predictor of tourism support, and perceptions of personal and social benefits of tourism positively influence support levels. Notably, tourism acceptance showed a mediating effect between tourism knowledge and support. These findings suggest the importance of resident education programs and the necessity of community participation in the initial stages of tourism development. Vargas-Sánchez et al. (115) confirmed significant differences in resident attitudes according to the destination's development stage. Specifically, the mediating effect of tourism acceptance varied by development stage, and residents' levels of tourism knowledge significantly influenced attitude formation. This extends Butler's tourism area life cycle theory and validates the dynamic nature of resident attitudes, suggesting the need for differentiated resident policies according to the destination's development stage.

Furthermore, Gursoy and Rutherford (116) found that perceptions of economic, sociocultural, and environmental impacts of tourism significantly influence tourism attitude formation, with tourism acceptance mediating this process. Nunkoo and Ramkissoon (117) confirmed that higher perceptions of tourism's positive impacts increased support for tourism, with tourism acceptance mediating this relationship. Notably, due to the characteristics of marine tourism destinations, the mediating effect of acceptance was more pronounced due to frequent resident–tourist interactions. Látková and Vogt (118) demonstrated that perceptions of tourism's community impact not only directly influence tourism attitudes but also indirectly affect them through tourism acceptance. Stylidis et al. (63) found that positive perceptions of economic and sociocultural impacts significantly influenced tourism attitudes, with tourism acceptance showing a mediating effect that strengthened these relationships. These previous studies consistently demonstrate that residents' tourism knowledge significantly influences tourism attitude formation, with tourism acceptance functioning as a key mediating variable in this process.

Fourth, place identity was found to have a positive moderating effect on the relationship between residents' tourism knowledge, destination perception, and tourism attitudes in marine sports tourism destinations. In this regard, Wang and Xu (51) found that residents with higher place identity demonstrated a stronger positive relationship between tourism knowledge and tourism attitudes. Specifically, residents with higher levels of pride and attachment to their region were found to form more positive tourism attitudes based on their tourism knowledge. Nunkoo and Gursoy (32) support these findings, showing that residents with higher place identity exhibit stronger relationships between perceptions of tourism's positive impacts and tourism attitudes. Stylidis (5) indicated that residents with higher place identity form more positive tourism attitudes based on their tourism knowledge and perceptions, with this tendency being particularly pronounced among residents who have higher pride in their local culture and traditions. Rasoolimanesh et al. (41) confirmed that residents of World Heritage tourism destinations with higher place identity form more favorable tourism attitudes based on their tourism knowledge and perceptions of tourism's positive impacts.

These previous studies consistently demonstrate that place identity has a significant moderating effect on the relationship between residents' tourism knowledge, tourism perception, and tourism attitudes in tourism destinations. Notably, residents with higher place identity were found to form more favorable tourism attitudes based on their tourism knowledge and positive tourism perceptions. This suggests that strengthening residents' place identity is a crucial policy imperative for the sustainable development of tourism destinations. These findings validate that place identity is not merely a psychological variable but rather a significant moderating variable that has substantial influence on the formation of residents' attitudes toward tourism.

## 7 Theoretical and practical implications

### 7.1 Theoretical implications

This study makes several significant contributions to the tourism literature. First, this study extends social exchange theory by demonstrating that in marine sports tourism destinations during post-pandemic recovery, the traditional cost–benefit evaluation framework must incorporate health risk perceptions and emotional safety considerations. Unlike conventional SET applications, our findings reveal that pandemic-induced psychological factors fundamentally alter how residents process tourism knowledge into attitudes, suggesting that crisis experiences introduce non-economic exchange dimensions that persist beyond the immediate threat period.

Second, the study establishes tourism acceptance as a theoretically distinct construct from tourism attitudes and support, positioned as a behavioral intention that mediates between cognitive evaluations and attitudinal outcomes. By operationalizing acceptance through three dimensions (welcoming tourists, interacting in daily life, and accepting community changes), this research contributes to the theoretical understanding of host–guest relationships in sport tourism contexts where physical proximity and shared resource use intensify interaction dynamics.

Third, our findings reveal that place identity functions as a dynamic moderator whose influence strengthens during crisis periods, particularly in specialized tourism contexts such as marine sports. This extends existing place identity theory by demonstrating that residents with stronger place attachment exhibit amplified knowledge–attitude relationships when their community's sporting identity is threatened, suggesting that crisis events activate protective mechanisms that intensify the role of place-based self-concepts in tourism attitude formation.

Fourth, the study advances sport tourism theory by incorporating activity-specific risk perceptions unique to marine sports (equipment dependency, weather vulnerability, skill requirements) into traditional resident attitude frameworks. This theoretical contribution highlights how the inherent risk characteristics of adventure sport tourism create additional layers of complexity in resident–tourist relationships that are absent in conventional tourism contexts.

Fifth, by examining residents who experienced marine sports tourism both before and during the pandemic, this research contributes to emerging post-crisis tourism theory by revealing that attitude formation processes are not static but evolve through distinct recovery phases. The findings suggest that the mediating and moderating mechanisms identified operate differently across crisis, immediate recovery, and normalization stages, necessitating temporally sensitive theoretical frameworks.

### 7.2 Practical implications

The findings offer valuable guidance for destination managers and policymakers in marine sports tourism destinations. First, destination management organizations should implement differentiated education curricula tailored to specific marine sports (surfing, diving, sailing) that address unique safety protocols, environmental impacts, and economic benefits. These programs should incorporate hands-on workshops where residents can experience marine sports activities, fostering empathy and understanding of tourist motivations while building community capacity to participate in tourism value chains through equipment rental, instruction, or guided tour services.

Second, tourism managers should establish a phased reintegration framework that gradually increases resident–tourist interactions based on community comfort levels and health metrics. This includes creating designated “tourism zones” with varying interaction intensities, implementing digital health passport systems for marine sports participants and establishing community feedback mechanisms that allow real-time adjustment of tourism volumes based on resident sentiment monitoring.

Third, destinations should co-create marine sports tourism products with residents that explicitly celebrate and preserve local maritime heritage, such as traditional fishing village tours combined with modern surfing experiences or historical maritime navigation workshops integrated with sailing activities. This approach should involve establishing resident advisory boards for all new marine sports infrastructure development and creating revenue-sharing mechanisms that directly link tourism success to community heritage preservation funds.

Fourth, tourism authorities must develop differentiated engagement approaches based on residents' business interests, with specific programs for (1) tourism-dependent residents focusing on service quality and sustainability, (2) non-tourism residents emphasizing indirect benefits such as infrastructure improvements and cultural exchange opportunities, and (3) tourism-skeptical residents providing platforms for voicing concerns and participating in tourism governance through citizen oversight committees.

Fifth, destinations should establish permanent crisis management frameworks that include (1) bi-monthly resident sentiment surveys with real-time dashboard visualization, (2) flexible tourism capacity adjustment mechanisms linked to community well-being indicators, (3) emergency communication protocols that prioritize resident health concerns while maintaining tourist confidence, and (4) economic support packages for residents affected by tourism fluctuations, funded through tourist taxes during peak periods.

## 8 Conclusion

### 8.1 Summary of findings

This study examined residents' attitudes toward marine sports tourism in the post-COVID-19 era through a comprehensive analysis of 231 residents who had lived in marine sports tourism destinations for >5 years. The research aimed to provide fundamental data for sustainable tourism development through enhanced community engagement. The empirical analysis yielded several significant findings.

First, residents' tourism knowledge demonstrated a positive influence on tourism attitudes (*β* = 0.331, *p* < 0.001), confirming that informed residents develop more favorable evaluations of tourism development. This relationship operated primarily through economic impact perceptions, suggesting knowledge enhances understanding of tourism's economic mechanisms.

Second, residents' perceptions of tourism showed a strong positive impact on tourism attitudes (*β* = 0.656, *p* < 0.001), emerging as the most powerful predictor in the model. The perception–attitude relationship explained 45.6% of the variance in tourism attitudes, highlighting the critical role of subjective evaluations in shaping overall tourism support.

Third, tourism acceptance exhibited significant mediating effects in both knowledge–attitude and perception–attitude relationships. For the knowledge pathway, tourism acceptance partially mediated the relationship (indirect effect = 0.192, 95% CI: 0.098–0.302), while for the perception pathway, the mediation was also partial (indirect effect = 0.168, 95% CI: 0.090–0.259). These findings confirm that behavioral willingness to welcome tourists serves as a critical mechanism linking cognitive evaluations to attitudinal outcomes.

Fourth, place identity demonstrated significant moderating effects, though in unexpected directions. Higher place identity weakened rather than strengthened the relationships between both knowledge and attitudes (interaction *β* = −0.147, *p* < 0.05) and perceptions and attitudes (interaction *β* = −0.183, *p* < 0.01). This suggests that strongly attached residents maintain consistent attitudes regardless of changing knowledge or perceptions, potentially reflecting protective responses to preserve community identity.

### 8.2 Limitations

This study contains several limitations that should be acknowledged. First, the cross-sectional design prevents causal inferences about the relationships among variables. While structural equation modeling suggests directional relationships, longitudinal research is needed to establish temporal precedence and examine how residents' attitudes evolve over time, particularly during different phases of post-pandemic recovery.

Second, the sampling approach presents constraints. The convenience sampling method, while enhanced through multichannel recruitment strategies, limits the generalizability of findings. Although efforts were made to maximize demographic diversity through strategic recruitment across different times and platforms, the self-selection inherent in voluntary online participation may have excluded certain resident groups, particularly those with limited digital access or lower engagement with community platforms. Additionally, the 5-year minimum residence requirement, while ensuring participants had sufficient experience with tourism impacts, may have excluded newer residents who could provide different perspectives on tourism development.

Third, the study's geographic focus on three marine sports destinations in Gangwon Province, South Korea, constrains external validity. Different marine sports tourism contexts—such as tropical diving destinations, Mediterranean sailing regions, or Pacific surfing communities—may exhibit distinct resident attitude patterns influenced by cultural, economic, and environmental factors not captured in this study.

Fourth, the reliance on self-reported measures introduces potential common method bias. Despite statistical tests suggesting this was not a major concern, residents' actual behaviors toward tourists may differ from their reported acceptance levels, particularly given the social desirability pressures in tourism-dependent communities. The use of a single online survey platform (Naver Forms) may have also introduced platform-specific biases.

Fifth, the study's timing during the late pandemic period (October–December 2024) captures a specific moment in tourism recovery that may not reflect stable, long-term attitude patterns. As communities fully emerge from pandemic restrictions and tourist volumes normalize, resident attitudes may shift in ways not anticipated by this research.

### 8.3 Future research directions

Building on this study's findings and addressing its limitations, several directions for future research emerge. First, longitudinal studies should track resident attitudes across different stages of tourism recovery, examining how the relationships among knowledge, perceptions, acceptance, and attitudes evolve as destinations transition from crisis to normalcy. Panel studies following the same residents over time would provide particular insights into attitude stability and change mechanisms.

Second, qualitative research methods, including in-depth interviews and ethnographic observations, should complement quantitative findings to understand the lived experiences behind statistical relationships. Residents' narratives about tourism acceptance, particularly those with high place identity who showed resistant attitude patterns, would illuminate the complex negotiations between community preservation and tourism development.

Third, comparative studies across different marine sports tourism contexts would enhance theoretical generalization. Research examining whether the mediating role of tourism acceptance and the moderating effect of place identity operate similarly across surfing destinations in Hawaii, diving locations in the Great Barrier Reef, and sailing centers in the Mediterranean would strengthen theoretical frameworks.

Fourth, experimental and quasi-experimental designs could establish causal relationships more definitively. Natural experiments occurring when destinations implement new tourism policies or experience external shocks provide opportunities to examine how resident attitudes respond to real-world interventions.

Fifth, the conceptualization and measurement of tourism acceptance requires further refinement. This study distinguished acceptance from general attitudes and political support, but future research should develop more nuanced measures capturing different dimensions of acceptance, such as economic tolerance, social welcoming, and cultural openness.

Sixth, investigation of boundary conditions for place identity's protective effect would advance theoretical understanding. Research should examine when place identity amplifies vs. attenuates tourism attitude formation, potentially identifying different types of place identity (e.g., traditional vs. progressive) that interact differently with tourism development.

Finally, practical intervention studies should test strategies for enhancing resident tourism acceptance while respecting place identity. Action research partnerships between academics and destination management organizations could develop and evaluate community engagement programs that foster sustainable tourism development through enhanced resident–tourist relationships.

In conclusion, this study provides valuable insights into the complex dynamics shaping residents' attitudes toward marine sports tourism in the post-pandemic era. By identifying tourism acceptance as a critical mediating mechanism and place identity as an important boundary condition, the research contributes to both theoretical understanding and practical management of sustainable tourism development in marine sports destinations.

## Data Availability

The raw data supporting the conclusions of this article will be made available by the authors, without undue reservation.
